# Mitotic phosphorylation activates hepatoma-derived growth factor as a mitogen

**DOI:** 10.1186/1471-2121-12-15

**Published:** 2011-04-13

**Authors:** Allen D Everett, Jun Yang, Monzur Rahman, Pratima Dulloor, David L Brautigan

**Affiliations:** 1 Department of Pediatrics, Cardiology Division, Johns Hopkins University, 600 N. Wolfe Street, Baltimore, 21287, USA; 2 Center for Cell Signaling and Department of Microbiology, University of Virginia, 1400 Jefferson Park Avenue, Charlottesville, 22908, USA

## Abstract

**Background:**

Hepatoma-derived growth factor (HDGF) is a nuclear protein that is a mitogen for a wide variety of cells. Mass spectrometry based methods have identified HDGF as a phosphoprotein without validation or a functional consequence of this post-translational modification.

**Results:**

We found that HDGF in primary mouse aortic vascular smooth muscle cells (VSMC) was phosphorylated. Wild type HDGF was phosphorylated in asynchronous cells and substitution of S103, S165 and S202 to alanine each demonstrated a decrease in HDGF phosphorylation. A phospho-S103 HDGF specific antibody was developed and demonstrated mitosis-specific phosphorylation. HDGF-S103A was not mitogenic and FACS analysis demonstrated a G2/M arrest in HDGF-S103A expressing cells, whereas cells expressing HDGF-S103D showed cell cycle progression. Nocodazole arrest increased S103 phosphorylation from 1.6% to 29% (P = 0.037).

**Conclusions:**

Thus, HDGF is a phosphoprotein and phosphorylation of S103 is mitosis related and required for its function as a mitogen. We speculate that cell cycle regulated phosphorylation of HDGF may play an important role in vascular cell proliferation.

## Background

HDGF [GenBank: NM_004494] is a heparin binding protein originally isolated from conditioned media of a human hepatoma (HuH-7) cell line. HDGF was subsequently shown to be a mitogen for many cell types with nuclear localization necessary for its mitogenic activity [1-6]. Expression of HDGF is developmentally regulated in at least the renal, cardiovascular and pulmonary systems [1,3,7] and re-expressed at least in the both the lung [8] and the arterial wall in response to injury [9], suggesting a role in tissue repair. HDGF has also been identified at least as an important prognostic marker in pathologic cell growth, as it is overexpressed in a number of cancers with expression linked to a poor outcome in lung, esophageal, pancreatic and hepatic cancer [10-13].

Many nuclear proteins undergo post-translational modification to regulate their activity. This is most clearly demonstrated by the cell cycle regulatory cyclin and CDK proteins which undergo both phosphorylation and dephosphorylation to regulate their activity [reviewed in [14]]. Previously we had shown by two-dimensional gel electrophoresis that HDGF in human melanoma cells has multiple isoforms that migrated with the same mass in SDS but had different pI [15], suggesting post-translational modifications, such as phosphorylation. In addition, in a proteomic screen for phosphorylated nuclear proteins, HDGF was identified by mass spectrometry to have multiple phosphorylated serines [16,17]. Whether HDGF is indeed phosphorylated in vivo, and whether phosphorylation affects HDGF function are all unknown.

In the present study, we detail that HDGF is indeed a phosphoprotein, identify S103 as a significant phosphorylation site and demonstrate that phosphorylation of S103 plays a critical role in regulating HDGF mitogenic function.

## Methods

### Cell culture

HEK-293T, MDA-MB231 and COS-7 cells were obtained from ATCC (Manassas, VA). Low passage mouse primary aortic vascular smooth muscle cells (VSMC) were isolated as previously described [1] and all lines maintained in DMEM supplemented with 10% fetal bovine serum (Gibco) at 37°C in 5% CO_2_. For proliferation experiments VSMC were serum starved for 36 hours, then incubated overnight with BrdU (10 μM, Roche Diagnostics, Indianapolis, IN). For cell cycle arrest studies, MDA-MB231 cells were seeded at 10^5 ^cells/ml in 6 well dishes containing a cover slip and DMEM with 10% serum. After 8 h cells were left in serum free (0.5% serum) media for overnight. Next morning cells were re-stimulated with 10% FCS. After 8 h cells were treated with or without 200 nM nocodazole for next 16 h. Next morning cells were briefly washed with ice cold PBS and fixed with 4% formaldehyde in DPBS.

### Plasmids and transfections

Full length wild type rat HDGF was cloned in pK7-GFP and pKH3 (vectors were gifts of Ian Macara, University of Virginia) [4] and substitution of serine (S) 103, 165 and 202 to alanine (A) or aspartic acid (D) was done using PCR (QuickChange Site Directed Mutagenesis, Stratagene). 1 × 10^6 ^HEK-293T, COS-7 or VSMC cells were plated in 60 mm dishes and transfected the following day with 4 ug of plasmid DNA using calcium phosphate (ProFection Mammalian Transfection System-Calcium Phosphate, Promega, WI) or FuGene (Roche Applied Science) according to the manufacturers' recommendations.

### Fluorescent activated cell sorting

HEK-293T cells were transfected as above to express GFP or GFP-HDGF fusions. 36 hours after transfection cells were processed for cell cycle FACS analysis with gating for no GFP and GFP after the method of Schmid and Sakamoto [18] (Becton Dickinson FACSCalibur Dual Laser) using ModFit LT software (Verity Software, Topsham, ME). Cell cycle analysis was expressed as percent in G1, G2 and S. Each FACS analysis was performed in triplicate with the results pooled from 4-5 separate experiments.

### Antibodies and immunoblotting

Anti-phospho-S103-HDGF was generated by Biosource (Hopkington MA) using a synthetic phosphopeptide corresponding to amino acids 95-107 of human HDGF with an N-terminal cysteine (CVKASGYQS(pS)QKKS) for coupling to keyhole limpet hemocyanin.

Western blot analysis was performed as previously described [1,4,7]. Briefly, phosphorylated proteins were enriched from 4 × 10^6 ^COS-7 cells using the PhosphoProtein Purification Kit (Qiagen, Valencia, CA) following the manufacturers instructions. For immunoblot analysis, COS-7 whole cell lysates and isolated proteins (20 μg) were separated by 10% SDS-PAGE and transferred to Trans-Blot Transfer Medium (Bio-Rad, Hercules, CA). Blots were blocked in TBS-T (0.1% Tween, w/v) and 5% bovine albumin (Roche) for one hour and probed with either anti-phospho-S103-HDGF (1:500) or anti-HDGF (1:1000) in TBS-T for 1 hour at room temperature. After washing with TBS-T membranes were incubated with an anti-rabbit secondary antibody coupled to horseradish peroxidase (1/30,000). After washing, the blots were developed using enhanced chemiluminescence (GE Healthcare).

### Immunocytochemistry

Immunocytochemical analysis was performed as previously described [1,4,7]. Briefly, COS-7 cells grown on glass coverslips in six well plates were fixed in 4% buffered paraformaldehyde for 30 minutes at room temperature then washed with cold PBS. Separate coverslips were incubated with the anti-HDGF (1:2000) or anti-phospho-S103-HDGF (1:250). Control coverslips were incubated with no primary antibody or preabsorbed primary antibody with 1 μg of the S103 phosphopeptide described above at the same concentration as the primary antibody. For BrdU detection, cells were fixed in 2% paraformaldehyde for 10 minutes at room temperature, with BrdU detected using a mouse monoclonal anti-BrdU antibody (6 ug/ml, Roche). Vector Red (Vector Laboratories) was used as a fluorescent substrate to identify specific HDGF or BrdU staining and DAPI as a specific DNA counterstain. Images were acquired on a Nikon Eclipse 400 microscope equipped with a MicroPublisher digital camera (Qimaging, Burnaby, BC, Canada) and merged using Adobe Photoshop cs software (Adobe Systems Inc., San Jose, CA). For nocodazole cell cycle arrest studies, MDA-MB231 cells were immunostained for anti-phospho-S103-HDGF and fluorescent microscope acquired images analyzed by Nikon NIS-element software. The total number of cells was counted by detecting size and intensity of DAPI staining. The number of phospho-S103 positive cells was identified as having at least 10 times more intense staining than non-treated control cells. Cells were counted from at least 3 different fields per coverslip for each experiment with a total of 3 individual experiments performed. Results were expressed as percent of phospho-S103-HDGF positive nuclei analyzed using a non-paired t-test with a P value of < 0.05 considered as significant.

## Results

### HDGF is a phosphoprotein

**Figure 1:**
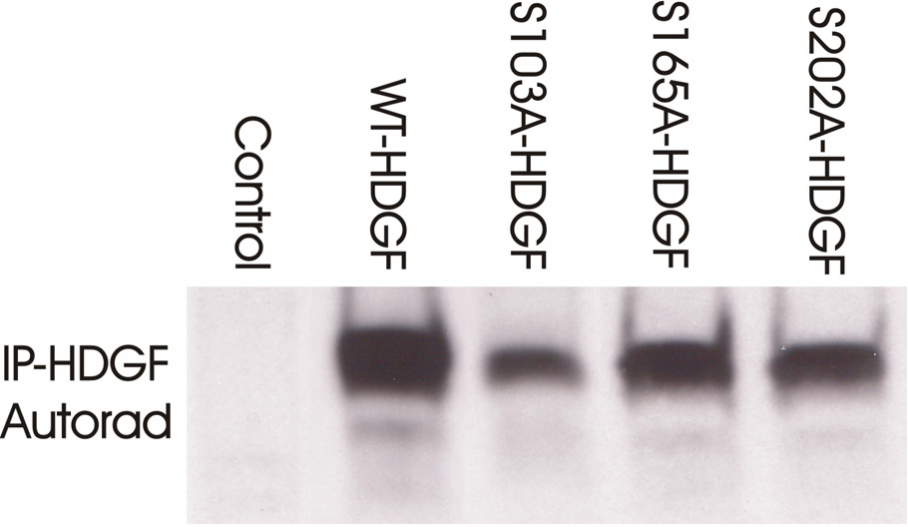
**HDGF is a phosphoprotein**. Autoradiogram of HDGF immunoprecipitated from COS-7 cells that transiently expressed wild type rat GFP-HDGF (WT-HDGF) or S103A, S165A, or S202A-HDGF substitution mutations after in vivo [^32^P] orthophosphate labeling for two hours, and resolution by SDS-PAGE.

Previously we found multiple forms of HDGF by 2D gels and suspected this was due to a post-translational modification such as phosphorylation that could change the pI of the protein. The NetPhosK 1.0 computer algorithm [19] using statistical ranking identified S103 as the most likely candidate site for phosphorylation (Score 0.86). In addition, S165 and S202 were recently identified as phosphorylation sites in HDGF by mass spectrometry of HeLa nuclear and HT-29 cell extracts [16,17]. Sequence comparisons confirmed that S103, S165 and S202 are conserved in mouse, rat and human HDGF. COS-7 cells were transfected to express GFP fusions of wild type HDGF or S103A, S165A or S202A substitution HDGF polypeptides and metabolically radiolabeled with [^32^P] orthophosphate for 2 hours. The tagged proteins were recovered by anti-GFP immunoprecipitation and as shown in Figure 1, HDGF wt was phosphorylated and phosphorylation of the S103A, S165A and S202A were reduced relative to wild type (Figure 1) demonstrating that all three of these serines were kinase substrates.

### HDGF S103 is phosphorylated during mitosis

**Figure 2:**
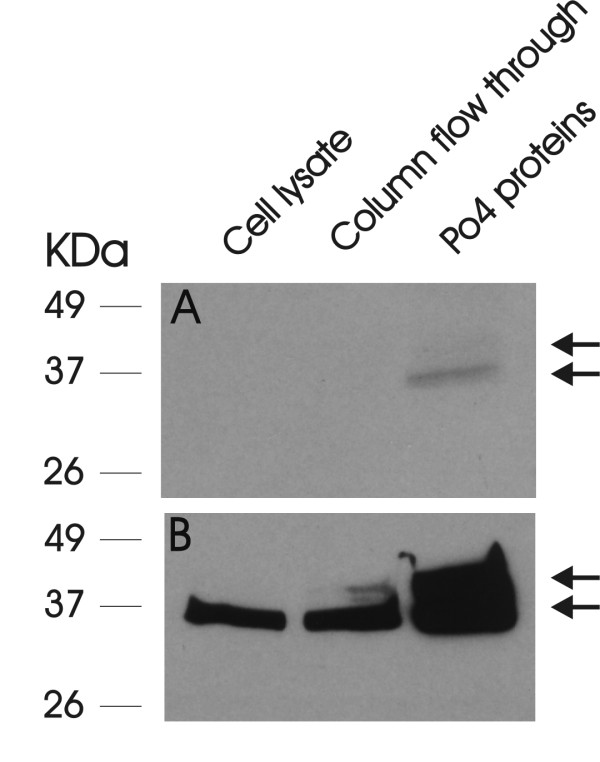
**Phospho-S103-HDGF Immunoblotting**. Western blot of COS-7 whole cell lysate (25 ug, Cell lysate), cell lysate PhosphoProtein column flow through (25 ug) or PhosphoProtein column bound proteins (25 ug, Po4 proteins, PhosphoProtein Purification Kit, see Methods) using a specific anti-phospho-S103-HDGF (top panel, A) or pan-anti-HDGF (bottom panel, B). Arrows = the high and low molecular weight bands in the Po4 protein lane with both antibodies.

**Figure 3:**
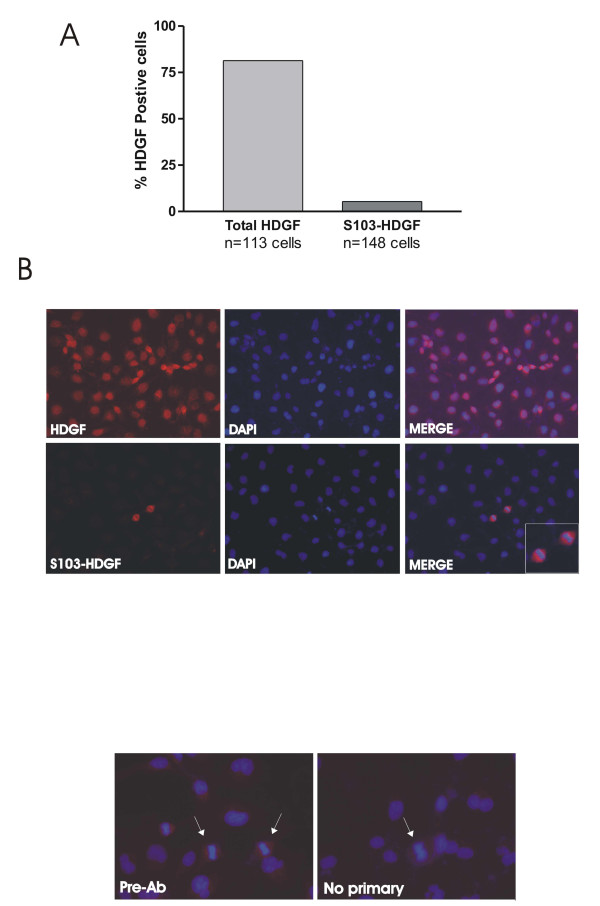
**Phospho-S103-HDGF expression in mitotic nuclei**. (A) COS-7 cells were immunostained for HDGF or phospho-S103-HDGF and percentage positive cells determined. (B) Immunostaining of COS-7 cells (red) for total HDGF and middle panel for phospho-S103-HDGF. (C) Staining specificity was proven by pre-absorbing the phospho-S103-HDGF antibody with the S103 phosphopeptide (Pre-Ab) or omission of the phopho-S103 HDGF antibody (No primary). Cells were counterstained with DAPI to identify the nucleus and DNA.

**Figure 4:**
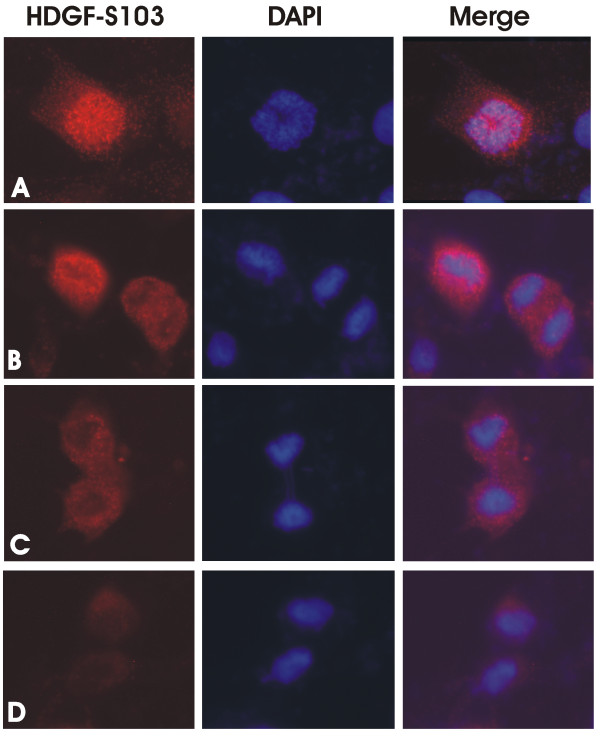
**HDGF S103 is phosphorylated during M phase of the cell cycle**. (A) Representative immunostaining for phospho-S103 HDGF was first detected in COS-7 cells that have undergone nuclear envelope breakdown and chromosomal condensation, (B) and in cells in metaphase and early anaphase. (C and D) HDGF-S103 phosphorylation rapidly falls to undetectable levels as daughter cells separate in late anaphase and telophase. Co-staining with DAPI demonstrates chromosomal DNA condensation in phospho-S103-HDGF immunostained cells (Merge).

**Figure 5:**
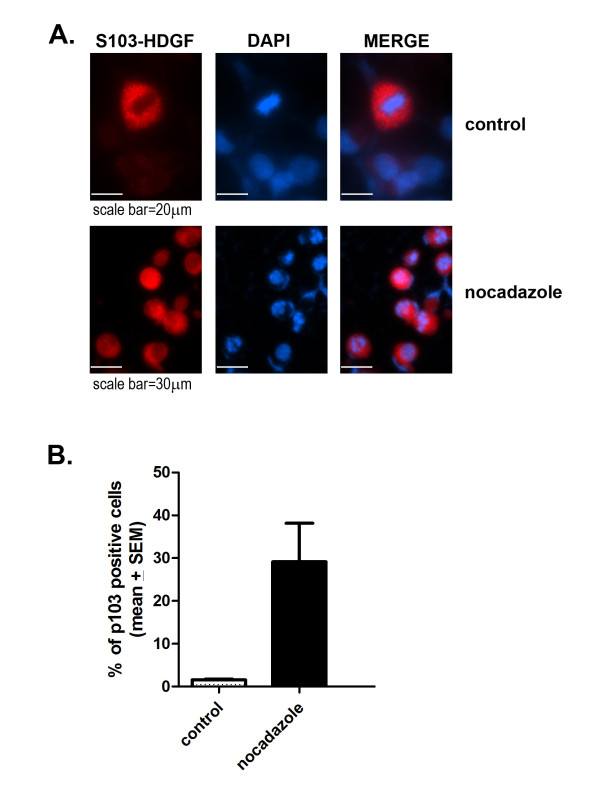
**Phosphorylation of S103 HDGF is increased during mitotic phase of cell division**. A. MDA-MB231 cells were treated without (control) or with nocodazole (200 nM) to arrest the cell cycle at mitosis and immunostained with the anti-phospho-S103-HDGF antibody and DAPI. B. Graph representing percentage of phospho-S103-HDGF positive cells in control and nocodazole treated cells (n = 3; p = 0.037 calculated by unpaired two-tailed T test).

To further explore HDGF S103 phosphorylation we developed a phospho-S103-HDGF antibody. Western blotting of enriched COS-7 phosphoproteins demonstrated detection of S103-HDGF phosphorylation (Figure 2). The phospho-S103-HDGF antibody identified both the high and low mass HDGF protein bands from the same COS-7 cell lysate detected with a wild type HDGF antibody [1]. With the phospho-S103 HDGF antibody, the higher mass HDGF band was less distinct compared to the lower mass band, but obvious with longer exposure. To map the expression of S103-HDGF, we immunostained COS-7 cells with both total and phospho-S103-HDGF antibodies (Figure 3A-C). Using the total HDGF antibody and COS-7 cells, HDGF was highly expressed in 81.4% of nuclei and with the phospho-S103-HDGF antibody, only 5.4% of cell nuclei were immuno-positive (Figure 3A). Of interest, the nuclei positive for phospho-S103-HDGF were all undergoing mitosis (Figure 3B), based on the condensed chromatin. Importantly preabsorbing the antibody with the target phosphopeptide, or the absence of the primary antibody in the immunostaining reaction demonstrated that staining was specific (Figure 3B). Further study (Figure 4) revealed that phospho-S103-HDGF was only detected during mitosis. Phospho-S103-HDGF was first detectable at the time of nuclear condensation and breakdown of the nuclear envelope, peaks at metaphase with alignment of chromosomes along the metaphase plate and disappears with daughter cell separation in anaphase. This was also evident when we cell cycle arrested cells with nocodazole and stained those cells with the phospho-S103-HDGF antibody (Figure 5). Nocodazole mitotic arrest significantly increased the number of phospho-S103-HDGF positive cells when compared to controls (29% vs 1.6%, P = 0.037, respectively).

### Phosphorylation of S103 is necessary for HDGF mitogenic function

**Figure 6:**
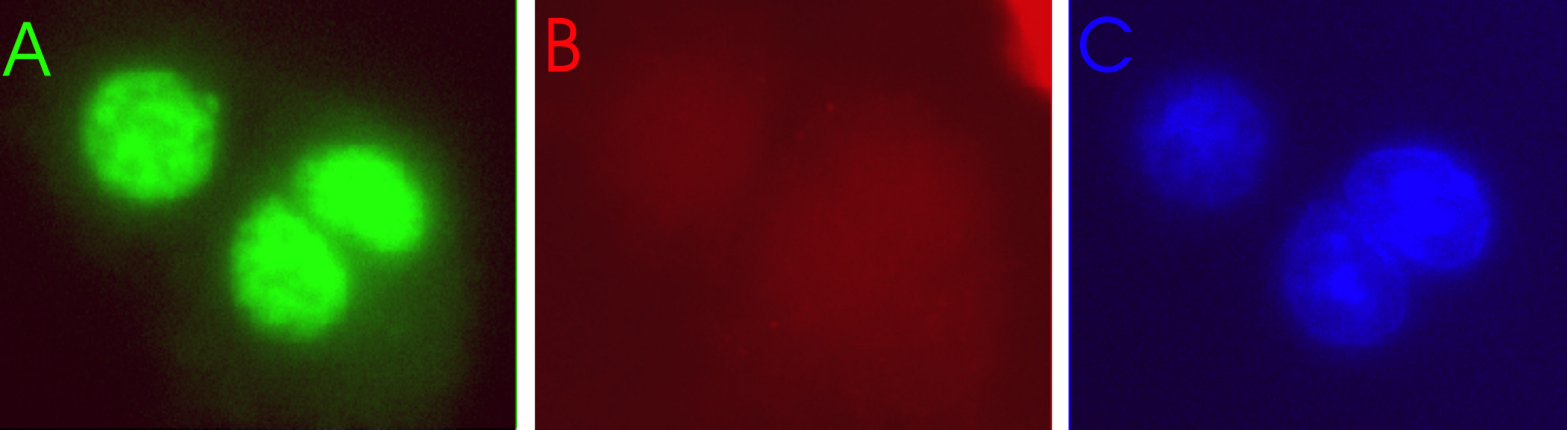
**HDGF-S103A is not a mitogen**. HDGF-GFP-S103A was expressed in mouse VSMC for 24 hours and cells labeled with BrdU overnight. Cells were immunostained for BrdU as a marker of DNA replication and observed for co-localization of GFP and BrdU. A = cells expressing HDGF-GFP-S103A, B = no staining for BrdU, C = DAPI staining to show that HDGF-GFP-S103A nuclear targeting is not affected.

HDGF is a potent mitogen for VSMC [1]. To examine the role of HDGF S103 phosphorylation in function as a mitogen, mouse VSMC were transfected to express GFP-HDGF S103A. Cells were serum starved for 24 hours then pulse-labeled with BrdU. BrdU incorporation (red) into DNA (blue) by transfected (green) cells was detected by fluorescent immunocytochemistry. As shown in Figure 6, HDGF-S103A traffics normally to the nucleus, demonstrating that S103 phosphorylation is not required for nuclear translocation or retention. However as shown in Figure 6, HDGF-S103A did not stimulate BrdU incorporation, showing it was not acting as a mitogen.

### Phosphorylation of HDGF S103 is necessary for cell cycle progression

**Figure 7:**
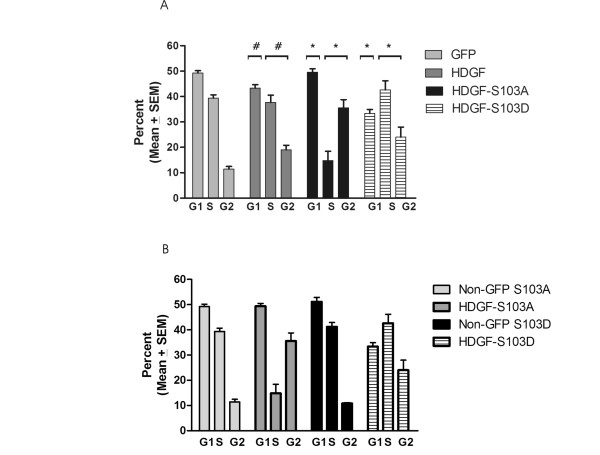
**Phosphorylation of HDGF S103 regulates its ability to stimulate cell proliferation**. A. HDGF-GFP, HDGF-GFP-S103A or HDGF-GFP-S103D fusion proteins were expressed in HEK-293 cells for 24 hours and the cell cycle analyzed in GFP and non-GFP expressing cells by FACS. (n = 4-5, with three replicates for each experiment.) B. Cell cycle analysis of cells expressing the HDGF fusions (GFP) vs non-GFP positive cells from the same experiments in A. # = HDGF vs GFP, * = HDGF-S103A or D vs HDGF. * and # = P ≤ 0.05.

To test for a possible role of phosphorylation in HDGF function, S103 was mutated to an aspartic acid (S103D) as a phospho-mimic residue. The effect of HDGF-S103D on cell proliferation was tested with asynchronous HEK-293 cells transfected to express, GFP fused to wild type HDGF, HDGF S103A or S103D and cell cycle analysis performed by FACS on the GFP positive cells and the GFP negative cells from the same sample as a control. As shown in Figure 7A, consistent with HDGF being a growth factor, HDGF significantly decreased the percentage of cells in G1 (43.3 ± 1.3% vs 49.6 + 3.0%, P < 0.009) and increased the percentage in G2 (19.0 ± 1.8% vs 11.4 ± 1.1%, P < 0.004) as compared to GFP. Unlike HDGF, HDGF S103A did not decrease the cell population in G1 (43.3 ± 1.3% and 49.6 ± 3.0% respectively), or increase the fraction of cells in S+G2 (56.7 ± 3.2 and 50.4 ± 3.0%, respectively). In contrast to HDGF S103A, HDGF S103D significantly decreased the fraction of cells in G1 (49.4 ± 3.0% and 33.4 ± 3.8%, respectively) and increased the fraction of cells in the S+G2 phase (50.4 ± 3.0% and 66.6 ± 3.8%, respectively). Therefore expression of HDGF S103D, a phospho-mimic, was a more potent mitogen than wild type HDGF and loss of phosphorylation on S103 abrogated HDGFs mitogenic function. This effect on the cell cycle was limited to transfected cells as non-transfected cells from the same plate demonstrated normal cell cycle proportions (Figure 7B). This demonstrates that the effects on the cell cycle were not transfection artifacts.

## Discussion

HDGF is an abundant nuclear protein with activity as a mitogen, in that it stimulates cell cycle progression. In this study we demonstrate phosphorylation of S103 during mitosis and show this phosphorylation is required for HDGF mitogenic activity. Our study of HDGF phosphorylation in vivo was suggested by the computer search engine NetPhosK 1 [19] that matches amino acid sequence to known protein kinase phosphorylation motifs, with statistical ranking for significance. This type of search engine is useful for identifying potential phosphorylation sites within a protein of interest. Separate studies had identified HDGF as a phosphorylated nuclear protein based on mass spectroscopy (MS) [16,17] or by in vitro kinase assays [20]. These studies indicated S132, S133, S165, T200 and S202 were phosphorylated in HeLa or HT-29 cells [16,17]. Our results identify S103 as a new, previously unknown significant HDGF phosphorylation site not previously identified by MS. Because S103 is only phosphorylated during mitosis, based on immunostaining with a phospho-amino acid specific antibody, this likely explains why pS103 was not found by MS in non-synchronized cells. This is supported by the relatively low levels of pS103-HDGF we observed by immunoblotting whole cell extracts. It is also unclear from these global MS studies whether the peptide containing S103 was detected. We demonstrate that S165 and S202 are also phosphorylated in vivo, but at possibly lower levels in COS-7 cells relative to S103, based on differences in radiolabeling of the mutated proteins. It is of interest that previously S165 had been predicted to be a Cdk2 substrate based on sequence, however mutation of S165 had no effect on the nuclear targeting of HDGF or on its mitogenic activity [4,5]. Although the kinase for S103 is not known, Salvi et al [20] have shown that HDGF can by phosphorylated in vitro by casein kinase 2. It is not known whether S132/133 are phosphorylated in vivo or whether S132/133 phosphorylation is functionally significant.

We found that phosphorylation of HDGF-S103 has a significant effect on HDGF mitogenic activity. A substitution mutation in HDGF to S103A to prevent phosphorylation nullified HDGF mitogenic activity, whereas a S103D phospho-mimic mutation was constitutively active, resulting in an increased mitogenic activity relative to wild type HDGF. This data would suggest that one model of VSMC proliferation is that activation of mitotic kinases results in phosphorylation of S103-HDGF, leading to increased cell proliferation. As the impact of the S103 mutants on the cell cycle was much more profound than the wild type protein, this would suggest that HDGF mitogenic function is dependent on phosphorylation and not just dependent on the amount of HDGF present.

Although the mechanism of phospho-S103-HDGF function during mitosis is unclear, it is of interest that another HDGF family member LEDGF, demonstrates metaphase chromatin binding, requiring cooperative interaction of the PWWP and AT-hook domains. Although HDGF does not contain AT-hook domains, it does bind DNA directly requiring a large 36 bp recognition sequence and requires the PWWP domain for DNA binding [21]. It is unclear how phosphorylation regulates this process either to induce a conformational change to increase binding or enhance binding with a chromatin binding protein. The HDGF PWWP domain was recently shown to dimerize on heparin and whether phosphorylation plays a role in potentially regulating HDGF dimerization on chromatin via the PWWP domain is an area of active research.

It is of great interest that a S282P mutation in the DNA methyltransferase 3b (DNMT3b, also a PWWP protein) gene results in the ICF syndrome (for immunodeficiency, centromeric instability, and facial anomalies) [22]. This serine is 4 amino acids carboxy to the PWWP domain in DNMT3b, and homologous to the location of S103 in HDGF. The conservation of this serine in relation to the PWWP domain and its mutation associated with a human disease, strongly implicates these serines in the function of PWWP proteins.

## Conclusion

HDGF is a mitotic phosphoprotein and phosphorylation of S103 plays an important role in regulating the proliferation of cells and the mitogenic function of HDGF.

## Authors' contributions

ADE conceived the experiments and wrote the manuscript. JY made the phospho HDGF mutants, generated the in vitro phosphorylation data and drafted that experimental section. MR performed nocodazole and cell sorting experiments and drafted the experimental results. PD performed cell transfections and immunohistochemical analyses. DLB edited the draft and contributed significantly to experimental design. All authors have read and approved the final manuscript.
